# HOW CAN DYING, DEATH, LOSS AND BEREAVEMENT BE INCORPORATED IN THE POLICY AND PRACTICE OF AGING IN PLACE?

**DOI:** 10.1093/geroni/igad104.0388

**Published:** 2023-12-21

**Authors:** Sarah Dury, An-Sofie Smetcoren

## Abstract

‘Aging in place’ has been one of the key goals for policymakers in the last 20 years, often translated into policies of deinstitutionalization. Due to the high costs of residential care, the home has re-emerged as a key site for the provision and consumption of care and support. Several critiques of this evolution have been formulated, including the challenges ageing in place might represent for older people, leading to reduced quality of life, and the need to consider low quality of older people’s houses, meaning that older people’s homes might not be the “best” places to age. However, what has received too little attention in the whole “aging in place” discourse is the end-of-life. This symposium addresses this by illustrating that we cannot talk about ageing in place without talking about dying in place. The authors present research on how dying, death, loss and bereavement might be included in the ageing-in-place policy and practice: Doran sets the stage with a critical reflection of ageing in place and the need to reflect on joining up ageing in place and end-of-life theory and practice. Smetcoren reports on the development of a ‘maison de mourance’ in a ‘co-housing and co-caring’ project in Brussels. Dury discusses consolation spaces in Flanders and how they decrease the taboo on death, grief and loss in the neighborhood. And Stegen reports on the caring neighbourhood-movement in Belgium and how some orient themselves to becoming part of a worldwide network of ‘compassionate communities’.

